# A Reproducible MRI‐Based Pipeline for Longitudinal Tracking of Laser‐Assisted Bioprinted Cells in Three‐Dimensional Constructs

**DOI:** 10.1002/nbm.70396

**Published:** 2026-09-10

**Authors:** Mina Medojević, Charles Handschin, Ayako Washio, Malou Lea, Laurence Dallet, Aleksandar Jakovljević, Emeline J. Ribot, Olivia Kérourédan

**Affiliations:** ^1^ INSERM, BioTis, Univ. Bordeaux Bordeaux France; ^2^ Department of Pathophysiology, School of Dental Medicine University of Belgrade Belgrade Serbia; ^3^ Implant‐Research Center, School of Dental Medicine University of Belgrade Belgrade Serbia; ^4^ Univ. Bordeaux, UFR des Sciences Odontologiques Bordeaux France; ^5^ INSERM U1026, ART BioPrint Bordeaux France; ^6^ Univ. Bordeaux, Tissue Bioengineering INSERM U1026 Bordeaux France; ^7^ Division of Endodontics and Restorative Dentistry, Department of Regenerative Science in Conservative Dentistry Kyushu Dental University Fukuoka Japan; ^8^ Univ. Bordeaux, CNRS, Plateforme d'Imagerie Biomédicale (pIBio), UAR3767 Bordeaux France; ^9^ Univ. Bordeaux, CNRS, Centre de Résonance Magnétique des Systèmes Biologiques (CRMSB), UMR5536 Bordeaux France; ^10^ CHU de Bordeaux, Pôle de Médecine et Chirurgie bucco‐dentaire Bordeaux France; ^11^ Centre de Compétence des Maladies Rares Orales et Dentaires, CCMR O‐Rares Bordeaux France; ^12^ Centre de Compétence des Maladies Osseuses Constitutionnelles, CCMR MOC Bordeaux France

**Keywords:** cell tracking, iron oxide labelling, laser‐assisted bioprinting, longitudinal monitoring, magnetic resonance imaging, tissue engineering

## Abstract

Laser‐assisted bioprinting (LAB) enables precise spatial patterning of living cells within three‐dimensional constructs; however, the lack of non‐destructive, standardised methods for longitudinal evaluation remains a major limitation to construct optimisation and translation. Magnetic resonance imaging (MRI) offers unique advantages for volumetric and repeated imaging due to its non‐invasiveness, yet its application to complex multilayer bioprinted constructs has not been systematically assessed using quantitative workflows. In this study, we developed and evaluated an MRI‐based pipeline for the longitudinal tracking and quantitative analysis of laser‐assisted bioprinted cells within three‐dimensional constructs of increasing architectural complexity. Iron oxide–labelled endothelial cells were bioprinted onto gelatin‐based biopapers in predefined patterns and assembled into constructs composed of three, six or nine stacked layers. Constructs were monitored at 4.7 T using a high‐resolution three‐dimensional steady‐state free precession sequence for up to 35 days, and quantitative image analysis was independently performed by three operators using the open‐source 3D Slicer image computing platform. MRI enabled clear visualisation of printed patterns and multilayer architectures over time due to the signal drop induced by iron oxide particles. Quantitative analysis demonstrated high inter‐operator reproducibility and revealed construct thickness–dependent differences in volume and signal‐to‐noise ratio. As a proof of concept, stacked bioprinted constructs were additionally visualised post‐mortem in a murine calvarial defect model, enabling discrimination of individual layers and printed patterns. Together, these results establish MRI as a robust, non‐destructive tool for the longitudinal evaluation of complex laser‐assisted bioprinted constructs.

## Graphical abstract

Laser‐assisted bioprinting was used to deposit iron oxide‐labelled endothelial cells in predefined patterns onto SCAP‐seeded gelatin biopapers to assemble multilayer constructs of increasing thickness. T2*‐weighted MRI monitored constructs for up to 35 days. Standardised analysis using an open‐source platform showed high inter‐operator reproducibility and revealed thickness‐dependent changes in quantitative MRI parameters, providing a robust, non‐destructive tool for longitudinal evaluation of complex bioprinted constructs.

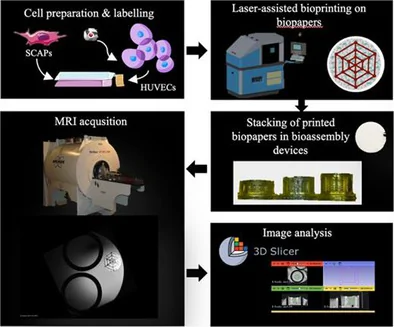

**Abbreviations**
CAD: computer‐aided design
CT: computed tomography
DCE: dynamic contrast‐enhanced
DLP: digital light processing
EGM‐2 MV: Microvascular Endothelial Cell Growth Medium‐2
FBS: foetal bovine serum
HUVEC: human umbilical vein endothelial cells
IOP: iron oxide particles
LAB: laser‐assisted bioprinting
MPIO: micron‐sized iron oxide particle
MRI: magnetic resonance imaging
OCT: optical coherent tomography
PBS: phosphate‐buffered saline
ROI: region of interest
SCAP: stem cell from the apical papilla
SNR: signal‐to‐noise ratio
SPIO: superparamagnetic iron oxide particles

## 1 Introduction

The regeneration of complex tissues remains a major challenge in regenerative medicine, requiring not only biologically active cells but also precise control over their spatial organisation, density and long‐term behaviour within three‐dimensional (3D) constructs [1]. In this context, bioprinting has emerged as a powerful approach for fabricating customised tissue constructs that recapitulate key structural and cellular features of native tissues [2]. Among the available techniques, laser‐assisted bioprinting (LAB) offers unique advantages, including high spatial resolution, nozzle‐free deposition and excellent cell viability, enabling the precise patterning of multiple cell types in predefined architectures [3, 4, 5, 6, 7]. LAB relies on the phenomenon of light‐induced forward transfer (LIFT), whereby laser interaction with a sacrificial layer propels bioink droplets onto a receiving substrate [8].

LAB has been successfully applied to engineer complex cellular arrangements and to investigate cell–cell interactions, migration and early tissue organisation [9, 10, 11]. However, as bioprinting strategies progress towards increasingly complex, multilayered and clinically relevant constructs, a major bottleneck persists: the lack of non‐destructive, quantitative and longitudinal methods to monitor printed cells within 3D architectures over time. Most commonly used evaluation techniques rely on endpoint analyses, such as histology or fluorescence microscopy, which require construct destruction and therefore prevent longitudinal follow‐up or direct comparison of construct evolution [12, 13, 14].

Magnetic resonance imaging (MRI) represents a particularly attractive solution to this limitation. As a non‐ionising, 3D imaging modality, MRI enables repeated examinations over extended periods while providing volumetric information on construct organisation [15]. Cellular MRI exploits the ability of cells to internalise contrast agents, most commonly superparamagnetic iron oxide particles (SPIOs), which induce signal loss on T2*‐weighted images through susceptibility effects [16, 17]. This approach has demonstrated high sensitivity, allowing detection of small cell populations and, under optimised conditions, even single labelled cells [18, 19]. LAB has previously been combined with cellular MRI to enable non‐destructive tracking of labelled cells after bioprinting. In this model, micron‐sized iron oxide particles (MPIOs)–labelled cells were bioprinted onto two‐dimensional biopapers both in vitro and in a mouse model of bone calvarial defect. T2*‐weighted MRI accurately captured the geometry of single‐layer printed patterns and enabled quantitative analysis of cell distribution. By optimising MPIO concentration and demonstrating a strong correlation between cell density and MRI signal, this work established MRI as a reliable tool for tracking laser‐bioprinted cell constructs in both in vitro and preclinical in vivo settings [5].

Despite these advances, several critical gaps remain. First, there is currently no standardised, reproducible pipeline linking bioprinting, MRI acquisition and quantitative image analysis, limiting objective comparison of constructs over time or across studies. Second, although construct thickness and architectural complexity are known to influence nutrient diffusion, mechanical stress and cell survival, their longitudinal impact on MRI‐derived quantitative parameters has not been systematically investigated. Finally, the translation of MRI‐based tracking approaches from in vitro constructs towards in vivo relevant configurations remains insufficiently explored.

Addressing these limitations is essential to move bioprinting from proof‐of‐concept demonstrations towards robust, comparable and translatable tissue engineering strategies. A standardised imaging and analysis framework would not only enable longitudinal monitoring of printed cells but also provide objective criteria to evaluate construct stability, viability and architectural performance.

Therefore, the aim of this study was to develop and evaluate a comprehensive MRI‐based pipeline for the longitudinal tracking and quantitative analysis of laser‐assisted bioprinted cells within 3D constructs of increasing complexity. Specifically, we sought (i) to assess the reproducibility of MR image analysis across independent operators using an open‐source platform; (ii) to investigate the temporal evolution of the MRI signal and labelled cells distribution, quantified through their volume and signal‐to‐noise ratio (SNR), in constructs composed of different numbers of stacked bioprinted layers; and (iii) to demonstrate the feasibility of this approach in a post‐mortem calvarial defect model as a first step towards translational applications.

By providing a reproducible, non‐destructive framework for monitoring bioprinted constructs over time, this work aims to bridge a critical methodological gap between advanced bioprinting technologies and their quantitative, longitudinal evaluation.

## 2 Experimental

### 2.1 Cell Culture

Stem cells from the apical papilla (SCAPs) and human umbilical vein endothelial cells (HUVECs) were used in this study. SCAPs were isolated from third molar germs obtained from young patients treated at the Oral Surgery Department of the Bordeaux University Hospital (CHU de Bordeaux). All procedures were approved by the relevant ethical authorities (Ministerial approval ‘DC‐2008‐412’; INSERM–CHU de Bordeaux agreement), and oral informed consent was obtained from all donors or their legal guardians. Samples were anonymised prior to use.

SCAPs were cultured in α‐minimum essential medium (α‐MEM; Gibco, Paisley, UK) supplemented with 10% foetal bovine serum (FBS, D. Dutscher, Bernolsheim, France) under standard conditions (37°C, 5% CO_2_, 95% relative humidity). HUVECs were isolated as previously described [20] and cultured in endothelial growth medium (EBM‐2 Basal Medium (CC‐3156) supplemented with EGM‐2 MV Microvascular Endothelial Cell Growth Medium SingleQuots supplements [CC‐4147], from Lonza Ltd., Basel, Switzerland). Endothelial cells were transduced using an integrative lentiviral vector expressing TurboRFP under the EF1a promoter from Vectalys, Toulouse, France, at a multiplicity of infection of 15 to enable fluorescent labelling. Cells between passages 6 and 10 were used for all experiments.

### 2.2 Cell Labelling With Iron Oxide Particles

RFP‐expressing HUVECs were labelled with Synomag‐D 70 nanoparticles (micromod Partikeltechnologie GmbH, Rostock, Germany; cat. no. 104‐00‐701; hydrodynamic diameter 70 nm, 20 mg Fe/mL in H_2_O) according to an established protocol [5]. Briefly, cells were incubated with SPIOs at a concentration of 1 mg/mL, washed three times with phosphate‐buffered saline (PBS), detached using trypsin–EDTA and centrifuged twice at 1000 rpm for 5 min to remove non‐internalised particles. Labelled cells were immediately used for bioink preparation and LAB.

### 2.3 Bioink Preparation

Following labelling, HUVECs were resuspended in EGM‐2 MV medium and concentrated to a density of 7 × 10^7^ cells/mL through successive centrifugation steps. The resulting cell suspension was used as bioink on the day of preparation to ensure optimal cell viability.

### 2.4 Biopaper Fabrication and Cell Seeding

Cross‐linked and lyophilised gelatin sheets with controlled porosity were used as biopapers, which were prepared according to the methodology previously described [21].

Biopapers were cut into 8‐mm discs, sterilised with ethylene oxide for 12 h and degassed under vacuum for 1 week. Prior to cell seeding, biopapers were placed in 48‐well plates and pre‐swelled in α‐MEM for 30 min. SCAPs were seeded at a density of 2 × 10^6^ cells/mL by depositing 50 μL of cell suspension onto each biopaper. After 30 min of incubation, an additional 450 μL of medium was added, and constructs were cultured for 4 days before bioprinting.

### 2.5 LAB

LAB was performed using a previously described LAB workstation [22].

In brief, Modulab printer (Inserm/Alphanov) was used for all of the experiments, configured with a nanosecond laser (Onda, BrightSolutions; 1064 nm, 2 ns pulse duration), operating at a rate of 100 kHz and an energy of 10 μJ. The donor substrate consisted of a quartz slide coated with a 20‐nm gold absorbing layer. The distance between donor and receiving substrates was set to 1000 μm.

**FIGURE 1:**
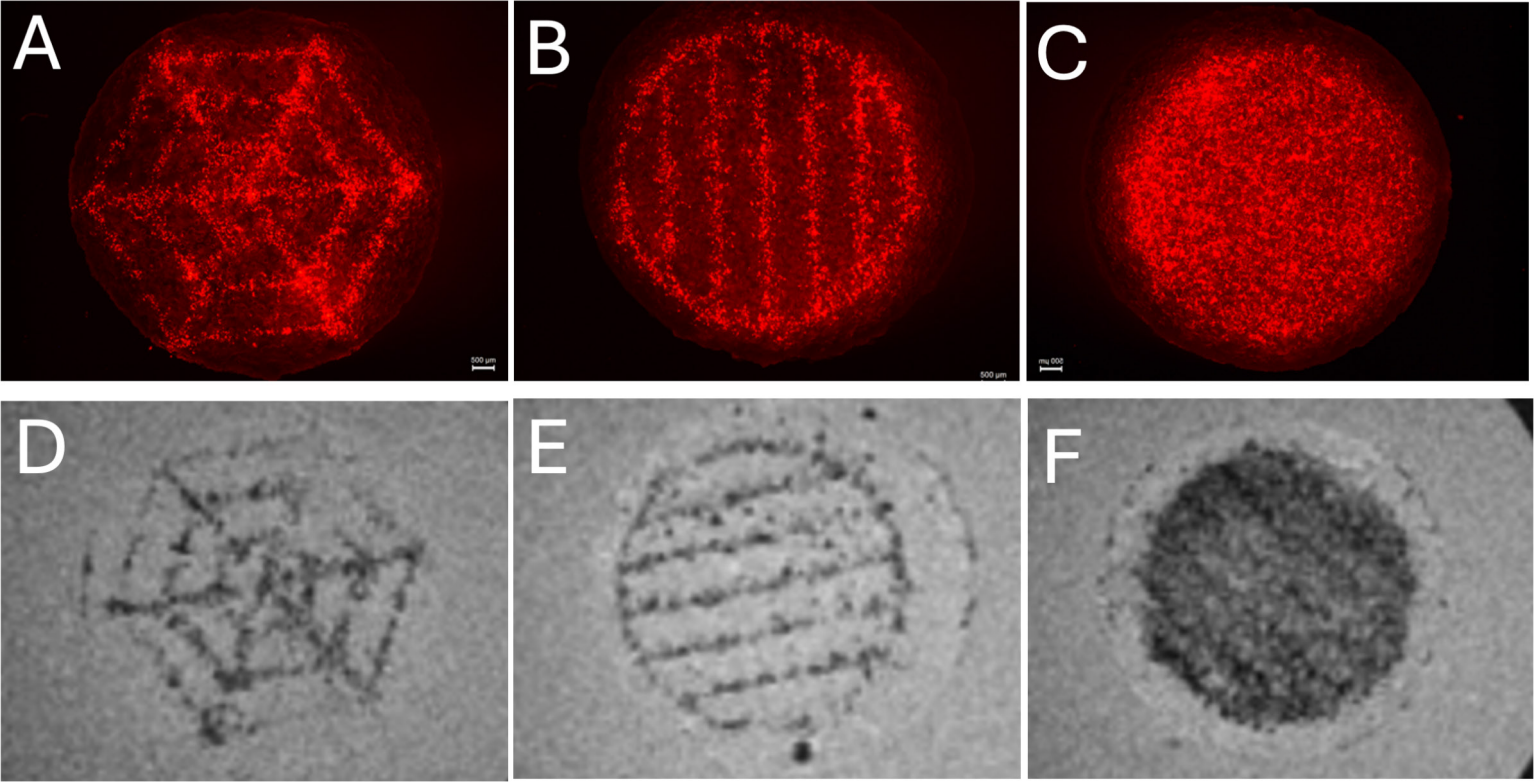
Multimodal imaging of individual biopapers printed with patterned cell distributions. (A–C) Fluorescence microscopy images of biopapers containing RFP‐expressing cells printed in distinct geometrical patterns: (A) spider web, (B) disc with parallel lines and (C) homogeneous full disc. (D–F) Corresponding MR images of the same biopapers incorporating SPIO‐labelled cells: (D) spider web, (E) disc with parallel lines and (F) homogeneous full disc.

Printing was conducted in air at room temperature using galvanometric mirrors for beam scanning. Three predefined patterns were printed: a full disc, a disc with parallel lines and a spider‐web pattern (6 mm diameter). A fluorescence binocular microscope equipped with a Leica DFC450 C digital camera and controlled by LAS V4.9 software (Leica Microsystems, Wetzlar, Germany) was used to assess the accuracy of the printed patterns (Figure 1A–C).

### 2.6 Assembly of Multilayer Constructs

Following bioprinting, biopapers were assembled into 3D constructs composed of three (S3), six (S6) or nine (S9) layers using custom‐designed bioassembly devices. Devices were designed using computer‐aided design (CAD) software (Onshape, Product Development Platform, Boston, MA, USA) and fabricated by digital light processing (DLP) 3D printing. Each device consisted of a fenestrated cylindrical structure with an inner diameter of 8.2 mm and an outer diameter of 10 mm, resting on a 12 mm diameter base, allowing precise alignment and stacking of biopapers while ensuring medium perfusion. Each window was separated from the next by a 1 mm space, whereas the windows themselves measured 0.55 mm in width and 2 mm in height. The cylinder height was 3, 4 or 6 mm, accommodating three, six or nine sheets, respectively. To ensure compatibility of the 3D model designed in Onshape with the selected printer, the file was formatted in STL format using Chitubox (SLA/DLP/LCD slicer software, Shenzhen, China). The bioassembly devices were printed using the Lumen X + DLP printer from Cellink (Volumetric Biotechnologies, Houston, TX, USA) and a suitable ink (PEGDA X, Volumetric Biotechnologies, Houston, TX, USA).

Constructs made of bioassembly devices and stacked layers of biopapers with interchanging order of patterns (full disc and circle with parallel lines) were placed in 35‐mm culture dishes containing 4 mL of co‐culture medium and maintained under standard culture conditions. Medium was refreshed twice weekly. MRI acquisitions were performed three times during the first week and weekly thereafter up to Day 35.

### 2.7 Post‐Mortem Calvarial Defect Model

As a proof of concept, three‐layer stacked bioprinted constructs were implanted post‐mortem into 7‐mm calvarial defects created along the sagittal suture in mice. Constructs were fixed in 4% paraformaldehyde prior to implantation. MRI imaging was subsequently performed to evaluate the visualisation of layers and printed patterns within an anatomically relevant environment.

### 2.8 Fluorescence Imaging

Imaging was performed using a Lumina III (PerkinElmer Inc., Boston, MA, USA).

For FRI experiments, excitation was performed at 745/30 nm, and fluorescence emission was detected with the 840/75 nm filter. Fluorescence images (1 s, 4 × 4 binning) and photographs (100 ms) were acquired successively. Fluorescence signals were analysed using Living Image software (Perkin Elmer).

### 2.9 MRI Acquisition

In vitro experiments were performed on a 4.7‐T Bruker Biospec system (Ettlingen, Germany) equipped with a gradient system capable of 660 mT/m maximum strength and 110 μs rise time. A circular surface coil (20 mm diameter, Doty Scientific) was used for signal excitation and reception. Two vials filled with water were placed below the coil for MR system adjustment, and a thin plate was placed above to precisely install a Petri dish containing the constructs of bioprinted cells, as already described by Kérourédan et al. [5].

For post‐mortem imaging, the animals were positioned in a 7‐T Bruker magnet, with the head at the centre of a four‐element (2 × 2) phased‐array surface cryoprobe. A volume resonator was used (75.4 mm inner diameter, active length 70 mm) for excitation.

All MR images were acquired using a 3D steady‐state free precession (SSFP) sequence in the coronal plane (i.e., parallel to the layers; the slice direction was oriented perpendicular to the layers). For the in vitro experiments, the following parameters were used: TE/TR = 4.5/10 ms, averages = 8; resolution = 130 × 130 × 104 μm for S6, S9B and S9C (leading to an acquisition time of 24 min 42 s), or resolution = 156 × 156 × 125 μm for S3 and S9A until and including Day 13 (leading to an acquisition time of 24 min 35 s) and resolution = 156 × 156 × 104 μm for Day 27 (leading to an acquisition time of 25 min 54 s). The same sequence was used for the post‐mortem experiments, with the following parameters: TE/TR = 2.8/5.6 ms, resolution = 97 × 55 × 62 μm, averages = 6 and acquisition time = 37 min.

### 2.10 Image Analysis

After acquiring the MR images, they were retrospectively corrected for signal bias caused by the surface coil (using the N4 option of the Advanced Normalisation Tools software). Then, image segmentation and quantitative analysis were performed using 3D Slicer, an open‐source software platform for medical image processing and visualisation (www.slicer.org). First, a region of interest (ROI) was drawn within the cell medium in which the holding device was submerged in to obtain the mean signal. To do so, the ROI was drawn on the axial plane, extending vertically from the bioassembly device from the bottom of the Petri dish to the top of the construct. The ROI included the average voxel count determined for each construct (mean ± SD = 344 ± 39). The ROI delineation was performed using the ‘Threshold’ option, with the mean signal of the medium used as the upper signal threshold, as iron‐labelled cells are known to produce hypointense signals on SSFP images [23]. A cropping step was then applied to preserve only the constructs within the holding device and the selected layers. This step was necessary because thresholded ROI also included the surrounding air present on the MR images. Cropping was first performed using the ‘Scissors’ tool within the ‘Operation: Erase outside’ and ‘Shape: Circle’ settings. ROIs were drawn on the coronal plane to isolate only the papers located inside the bioassembly device. Additional cuts were then applied on the axial and sagittal planes above and below the constructs using ‘Operation: Erase inside’ and ‘Shape: Rectangle’ to remove background noise. In order to prevent any bias due to the interface between air and the layers, the top ones were not included in the segmentations. The number of layers retained for analysis was selected to preserve the same construct ratio across samples. For the three‐, six‐ and nine‐layer constructs, two, four and six layers were retained, respectively, corresponding to a ratio of 0.66. The retained layers were manually selected by cutting the ROI of the labelled cells, using the ‘Scissors’ tool (‘Operation: Erase inside’) and a free‐form ROI shape encircling the corresponding layers. The number of voxels contained within this ROI and their corresponding signal intensity were measured. A ROI was also drawn in the background region of the image above the construct on three different axial slices (one slice containing the last part of the construct at each end and one slice passing through the center of the construct). This ROI contained an average voxel count of 1882 ± 161. The mean signal intensity and standard deviation of the noise were recorded to calculate the SNR of the labelled cells as follows:

$$\mathrm{SNR}=\frac{\text{Mean signal intensity of}\;\mathrm{ROI}\;\text{containing labelled cells}}{\text{Standard deviation of noise signal intensity}}$$

The ROI volume was calculated using the following equation:

ROIvolume=Voxel count×Δx×Δy×Δz

where Δx, Δy and Δz present the size of one voxel in x, y and z directions in μm.

Images were analysed by three independent operators, who were instructed not to modify the thresholding or ROI selection procedures according to the expected results or construct type.

The exact steps for data extraction using 3D Slicer are shown in Figure S1.

### 2.11 Data and Statistical Analysis

Statistical analyses were performed using one‐way or two‐way analysis of variance (ANOVA) in GraphPad Prism Version 10.6.1 (799) for Mac OS (GraphPad Software, Boston, MA, USA; www.graphpad.com). In total, five independently bioprinted multilayer constructs were analysed: one three‐layer construct (S3), one six‐layer construct (S6) and three nine‐layer constructs (S9A–S9C). For each time point, three independent operators performed the image analysis, yielding three separate measurements per construct and time point. Normality of the data was assessed using the Shapiro–Wilk test. For inter‐operators' variability, the coefficient of variation (CV, %) was calculated using the following equation for each construct and time point:

$$\text{Coefficient of variation}\;\mathrm{CV}\;\left(\%\right)=100\times \frac{\text{standard deviation}\;\left(\text{operator}\;1,\text{operator}\;2,\text{operator}\;3\right)}{\text{average}\;\left(\text{operator}\;1,\text{operator}\;2,\text{operator}\;3\right)}$$

ROI volume and SNR at Day 1 were compared between all constructs (*n*: S3 = 3, S6 = 3, S9 = 9) using one‐way ANOVA with repeated measures. Longitudinal tracking up to 28 days across all constructs was compared using a two‐way ANOVA with appropriate within‐subject factors, followed by Tukey's post hoc multiple‐comparison test. All tests were two‐tailed, and differences were considered statistically significant for *p* < 0.05.

## 3 Results

### 3.1 MRI Visualisation of Laser‐Assisted Bioprinted Patterns and Multilayer Constructs

Fluorescence microscopy confirmed successful printing of labelled cells onto individual biopapers in the three predefined patterns (spider web, disc with parallel lines and full disc). Corresponding MRI acquisitions enabled clear visualisation of the same patterns, demonstrating that labelled cells were readily detectable using MRI immediately after bioprinting, due to the internalised IOP (Figure 1).

**FIGURE 2:**
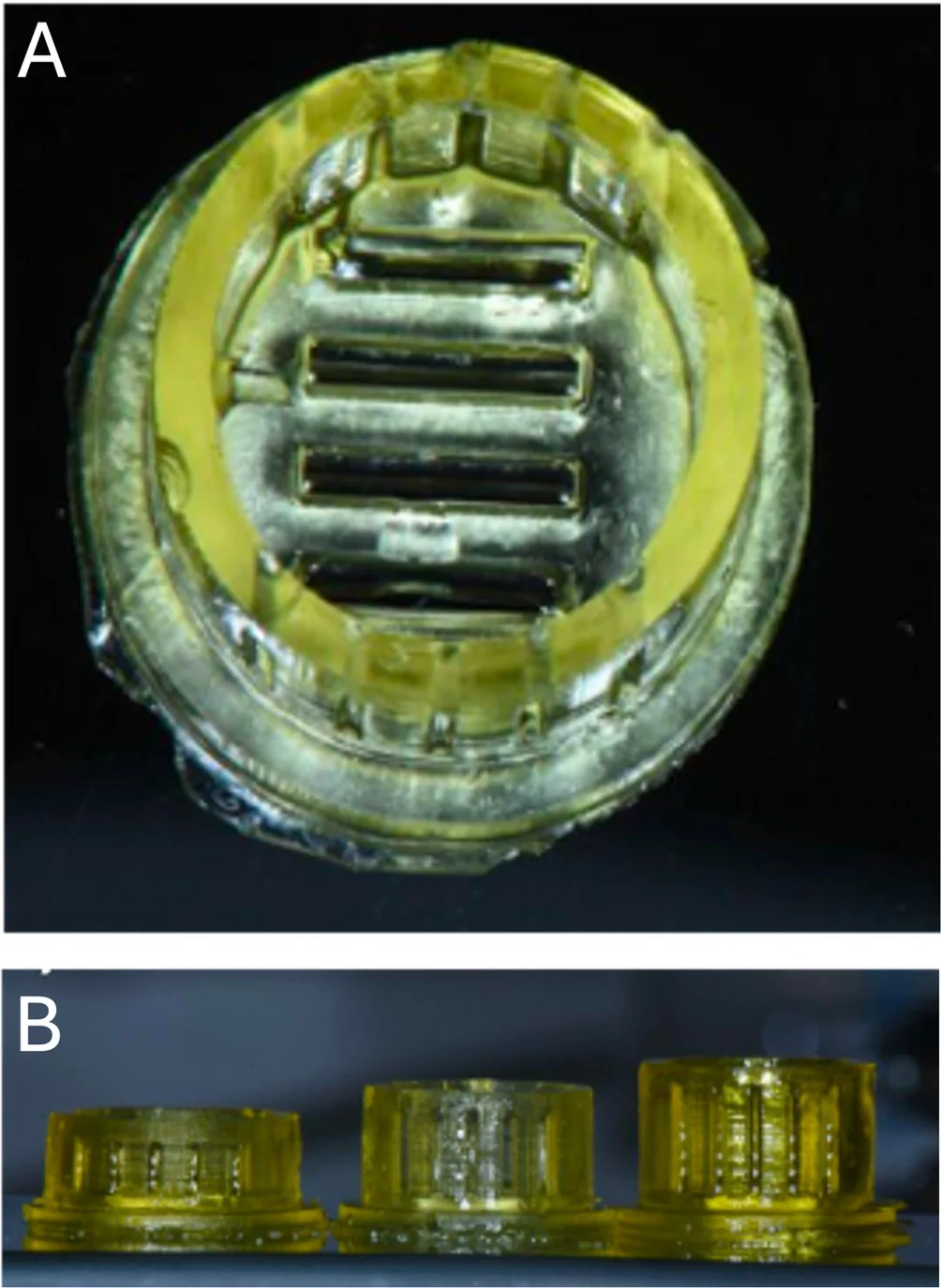
Design and modular configuration of the 3D‐printed assembly device. (A) Top view of the 3D‐printed assembly device. (B) Modular configurations of the device accommodating three (left), six (middle) and nine (right) stacked biopaper layers.

**FIGURE 3:**
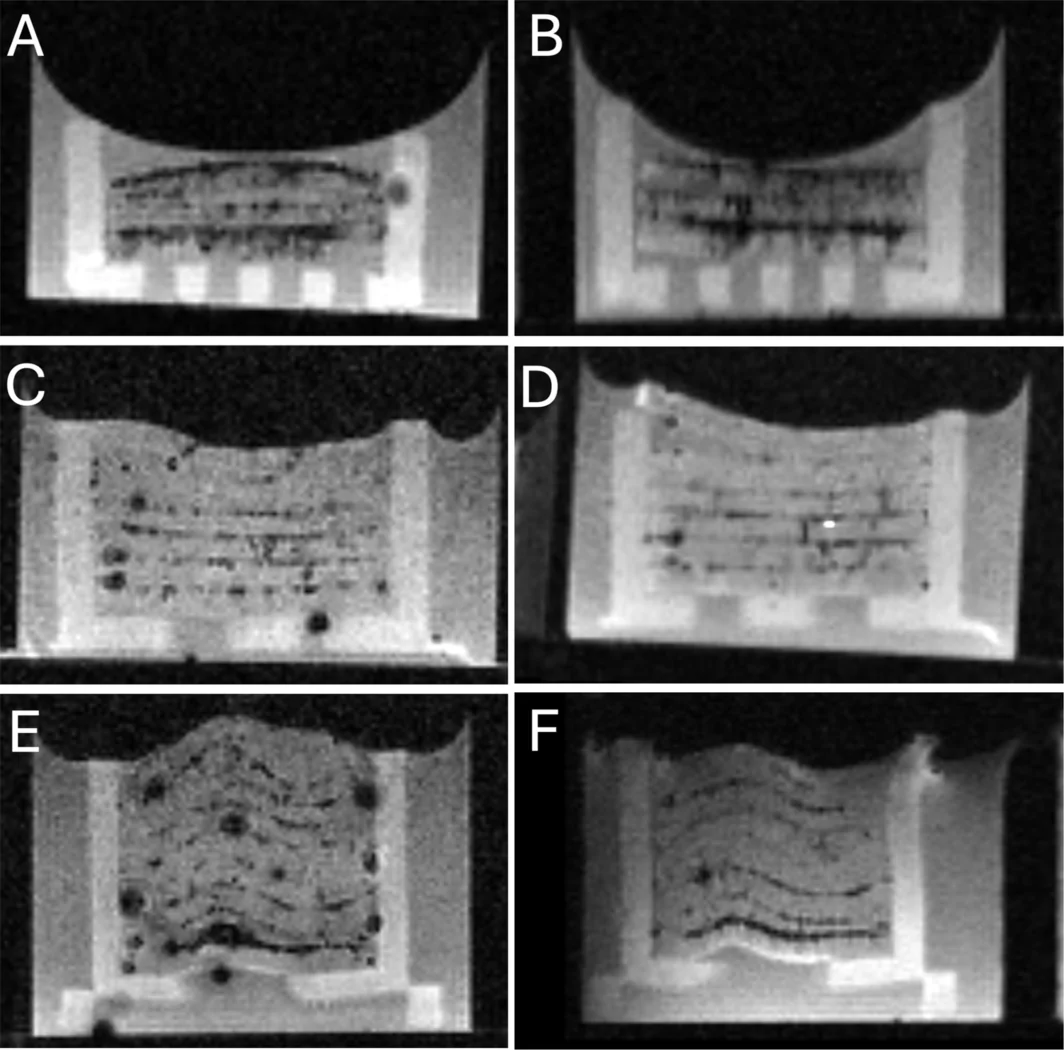
Longitudinal MRI assessment of multilayer biopaper constructs containing labelled cells. (A,B) MR images of the three‐layer construct acquired on Day 1 (A) and Day 28 (B). (C,D) MR images of the six‐layer construct acquired on Day 1 (C) and Day 28 (D). (E,F) MR images of nine‐layer constructs acquired on Day 1 (E) and Day 28 (F).

Following bioprinting, individual biopapers were assembled into 3D constructs composed of three, six or nine stacked layers using dedicated bioassembly devices (Figure 2). SSFP images enabled the detection of iron‐labelled cells as signal hypointensities, the bioassembly device in a hyperintense signal and the cell medium as a grey signal. MRI acquisitions performed longitudinally over 35 days enabled unambiguous identification of all stacked configurations, with clear detection of labelled cells across all layers and time points (Figure 3). Signal voids corresponding to iron‐labelled cells remained spatially consistent with the initial printed patterns, confirming MRI's ability to resolve multilayered architectures within 3D bioprinted constructs.

### 3.2 Reproducibility of MRI‐Based Quantitative Analysis Across Operators

To assess the robustness and reproducibility of the printing, the 3D building into constructs and the proposed MRI analysis pipeline, quantitative image analysis was independently performed by three operators using identical datasets. Two parameters were extracted for each construct: (i) the ROI volume, reflecting labelled‐cell spreading, and (ii) the SNR of the corresponding voxels contained within the ROI.

**FIGURE 4:**
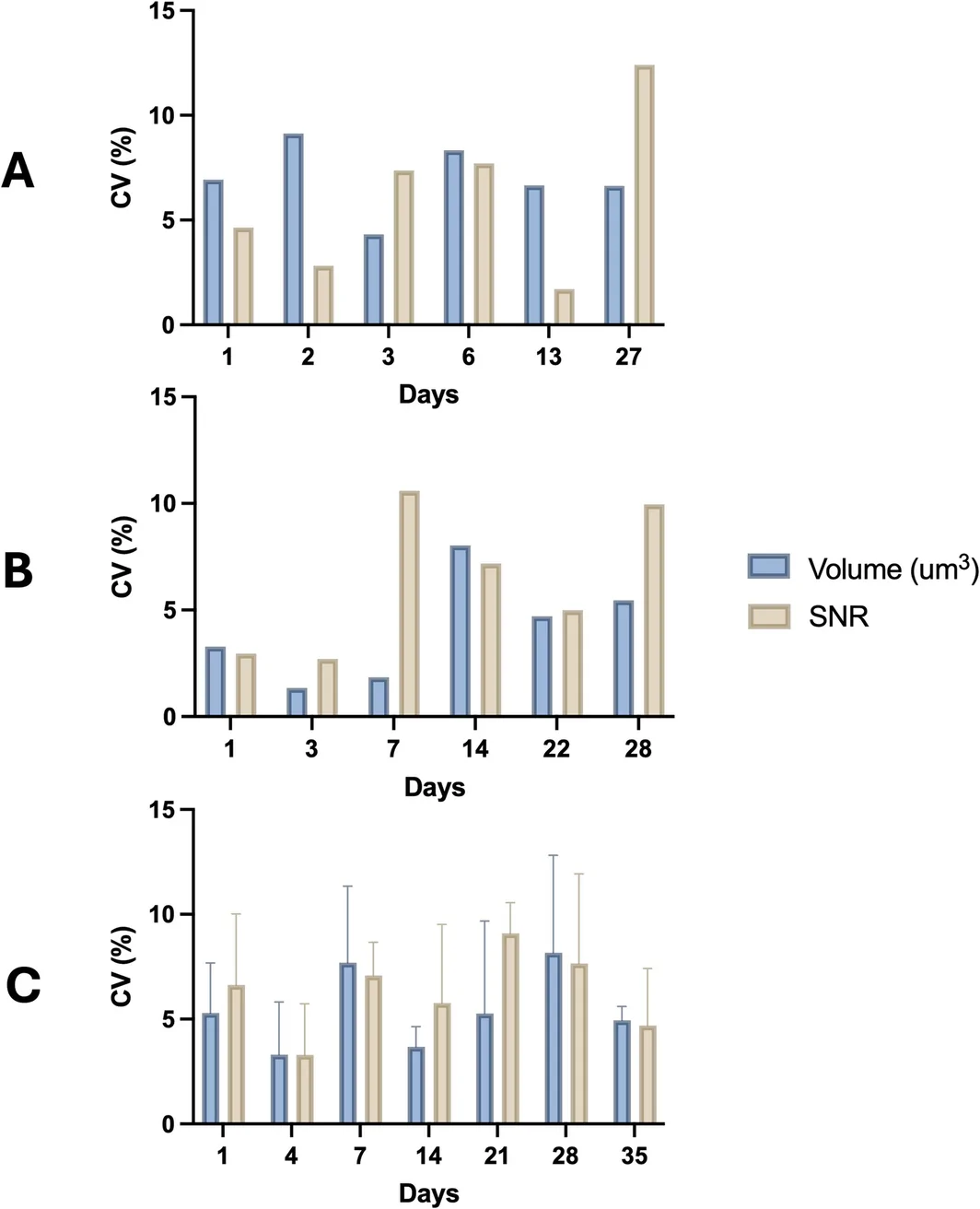
Coefficient of variation (CV, %) for volume and SNR values across three independent operators for constructs composed of three layers (A), six layers (B) and nine layers (C). The bars correspond to the mean values of volume and SNR across three independent operators, with standard deviation bars shown in graph C due to the study of multiple nine‐layer constructs (*n* = 3).

CV measurements yielded consistent results for volume and SNR, regardless of the number of layers within the constructs or the acquisition time points (Figure 4). Due to the high MR contrast between the labelled cells and the medium, less than 13% variability was observed, especially for volume. SNR values exhibited similar variability, never exceeding 12%.

### 3.3 Influence of Construct Thickness on MRI Signal Characteristics

**FIGURE 5:**
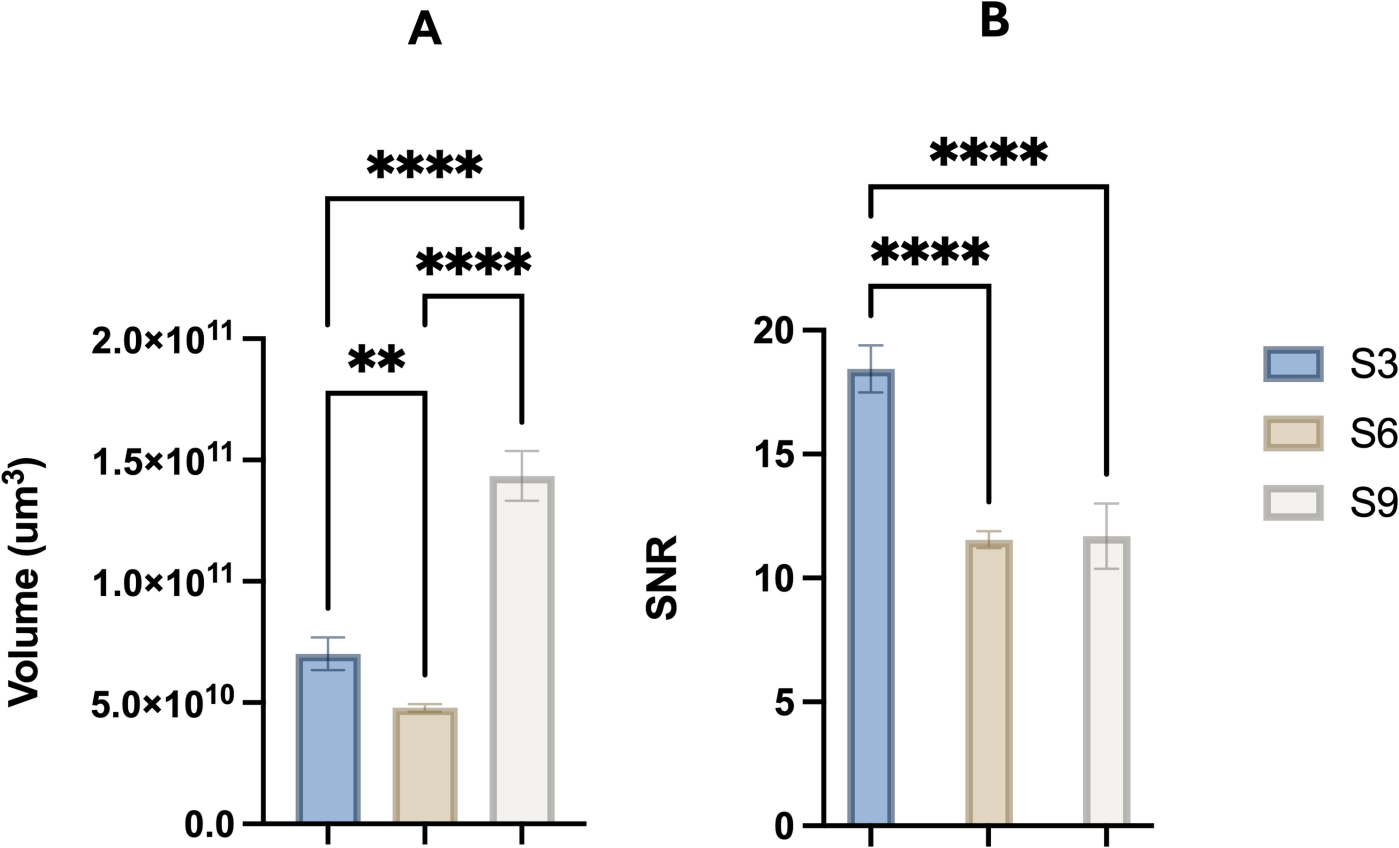
Volume (A) and signal‐to‐noise ratio (B) measured 1 day after bioprinting and assembly of three‐, six‐ and nine‐layer constructs (denoted S3 S6 and S9, respectively). Bars represent mean values measured across operators #1–#3. Statistical significance is indicated by asterisks (**p < 0.01, ****p < 0.0001).

The volume and SNR were measured 1 day after bioprinting and assembly of three‐, six‐ or nine‐layer constructs (Figure 5).

The volume containing iron‐labelled cells was approximately threefold larger in the nine‐layer constructs than in the six‐layer constructs and approximately twofold larger than in the three‐layer constructs. Interestingly, the volume measured in the six‐layer constructs was lower than that observed in the three‐layer constructs.

The SNR was higher in the three‐layer constructs, compared to the six‐ and nine‐layer constructs. No significant difference in SNR was detected between six‐ and nine‐layer constructs.

### 3.4 Longitudinal Evolution of MRI‐Derived Quantitative Parameters

**FIGURE 6:**
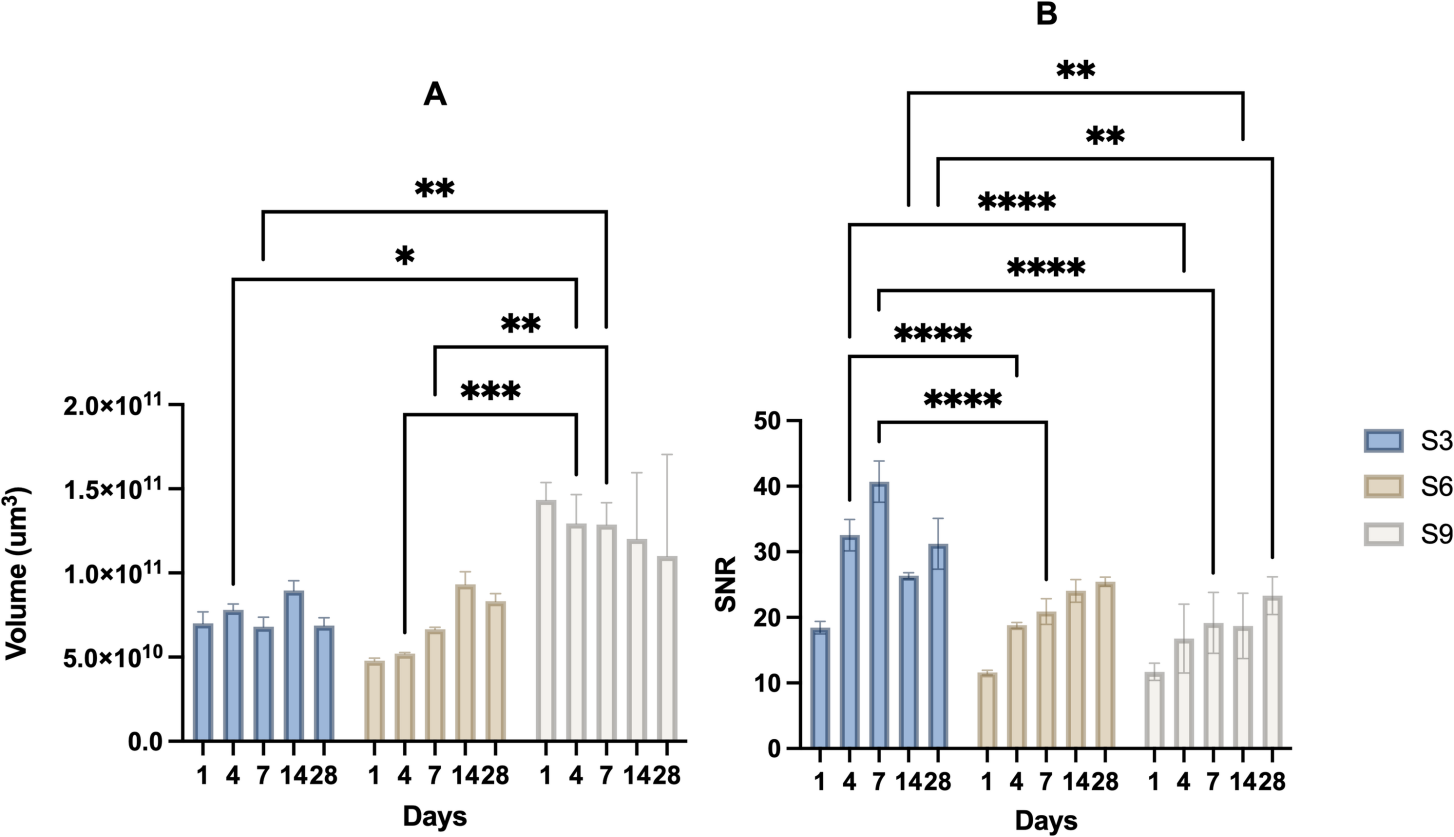
Longitudinal monitoring of volume (A) and signal‐to‐noise ratio (SNR) (B) in multilayer constructs composed of three, six and nine layers (denoted S3, S6 and S9, respectively). Lines represent mean values across operators #1–#3. Statistical significance is indicated by asterisk (*p < 0.05, **p < 0.01, ***p < 0.001, ****p < 0.0001).

The temporal evolution of volume and SNR was evaluated for each construct throughout the monitoring period. Across all construct configurations, the signal associated with labelled cells remained detectable throughout the experiment, enabling longitudinal follow‐up for up to 28 days (Figure 6).

For the three‐layer constructs, the volume remained stable, whereas the SNR values increased until Day 7 before unexpectedly decreasing at later time points. The six‐layer constructs showed increases in both volume and SNR values, whereas the nine‐layer constructs exhibited a decrease in volume associated with an increase in SNR.

### 3.5 Evolution of MRI‐Derived Quantitative Parameters for Three Different Nine‐Layer Constructs

**FIGURE 7:**
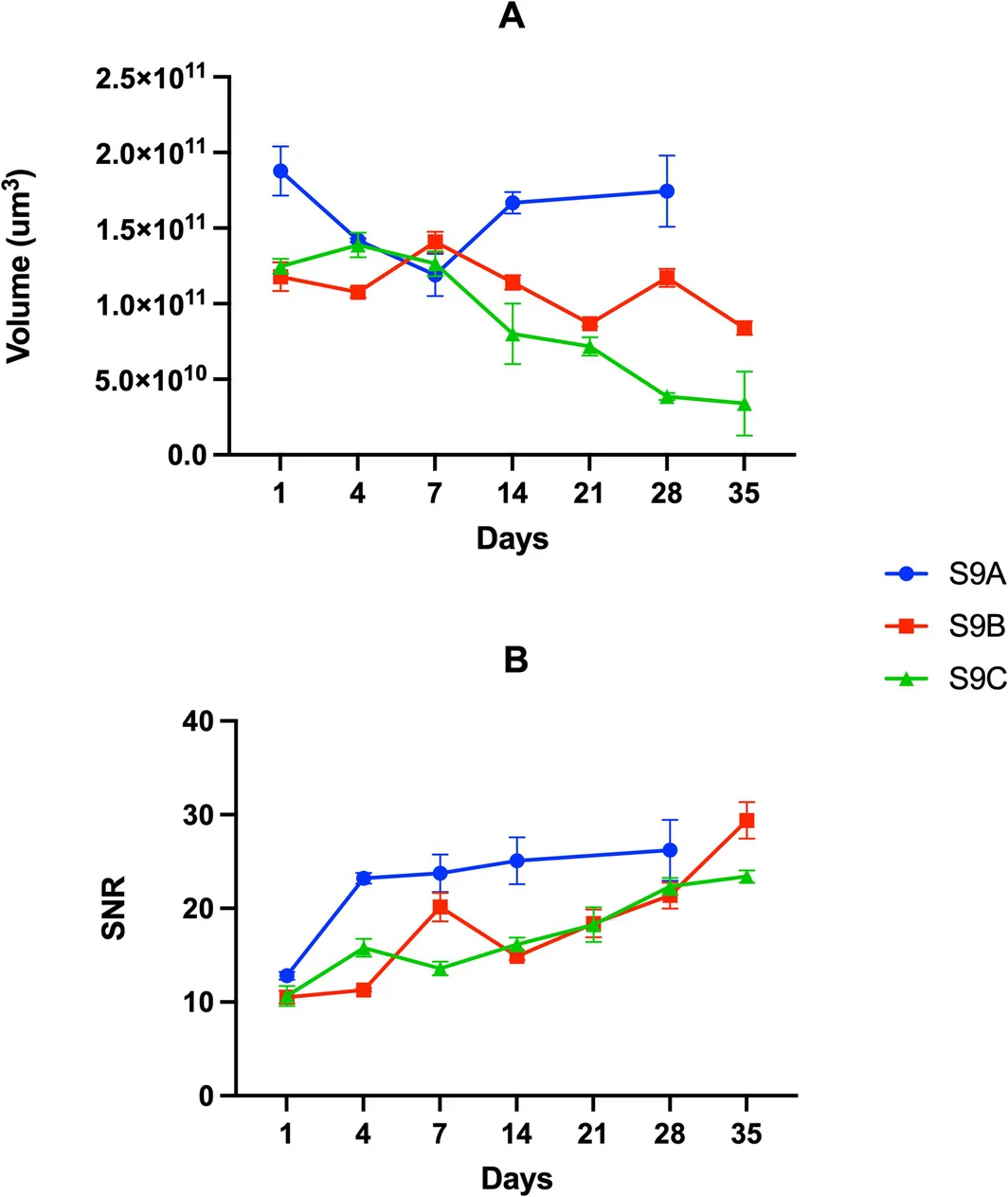
Temporal evolution of volume (A) and signal‐to‐noise ratio (SNR) (B) for three independent nine‐layer constructs (denoted S9A, S9B and S9C, respectively).

The temporal evolution of the ROI volume and SNR across the three nine‐layer constructs is shown in Figure 7. Whereas some nine‐layer constructs exhibited a progressive decrease in volume over time (S9B and S9C), S9A showed an initial decrease in volume until Day 7, followed by an increase to values comparable to Day 1.

In parallel, SNR values in S9B and S9C increased progressively over time, whereas the SNR of S9A doubled by Day 4 and remained stable thereafter.

### 3.6 Post‐Mortem MRI Visualisation of Stacked Bioprinted Constructs in a Calvarial Defect Model

**FIGURE 8:**
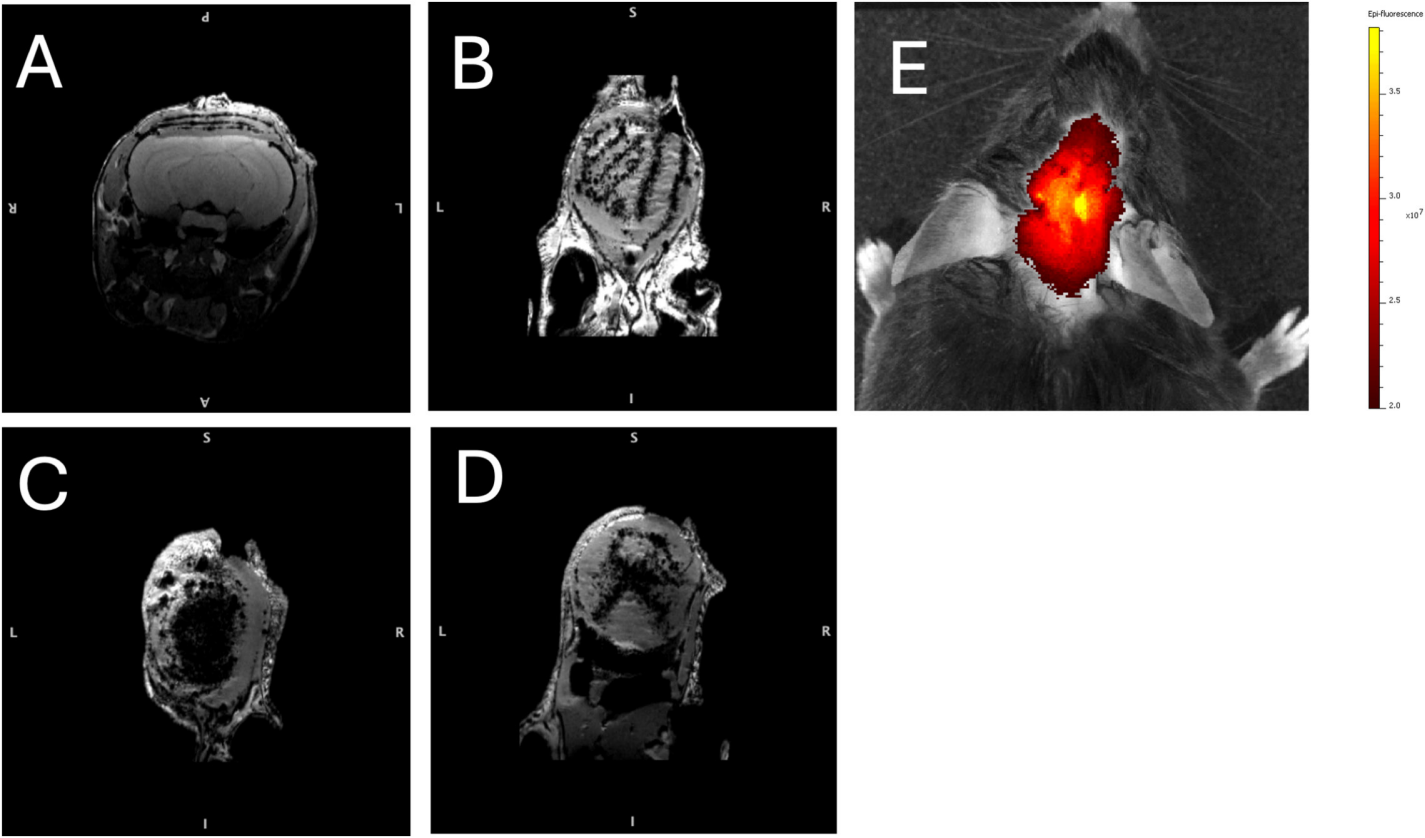
MR and fluorescence images of patterned bioprinted papers in calvarial bone defects in a representative mouse, acquired post‐mortem. (A) Axial MR image showing three distinct stacked bioprinted paper layers above the brain. (B–D) Coronal MR images of individual papers within the construct: (B) parallel lines, (C) homogeneous full disc and (D) spider web pattern. (E) Fluorescence image of the same mouse showing the signal intensity of RFP‐expressing bioprinted cells overlaid on an anatomical picture.

As a proof of concept for potential translational applications, a three‐layer stacked bioprinted construct was implanted post‐mortem into murine calvarial bone defects and imaged using high‐field MRI. SSFP images enabled clear visualisation of individual layers and discrimination between different printed patterns within the defect site (Figure 8).

MRI provided high‐resolution cross‐sectional images, allowing precise identification of each bioprinted layer, whereas the corresponding fluorescence imaging did not provide sufficient spatial resolution to resolve the different printed patterns (Figure 8).

## 4 Discussion

The present work addresses a critical methodological limitation in bioprinting research: the lack of non‐destructive, standardised approaches for the longitudinal evaluation of complex 3D constructs. Although a previous study demonstrated the feasibility of tracking laser‐assisted bioprinted cells using MRI, it was primarily limited to single‐layer constructs and qualitative assessments of printed patterns [5]. By extending cellular MRI to multilayer laser‐assisted bioprinted constructs and coupling it with a reproducible quantitative analysis workflow, the present study provides a framework enabling objective, longitudinal comparison of construct evolution as architectural complexity increases.

A first important outcome of this work is the demonstration of the robustness and reproducibility of the proposed MRI analysis pipeline, as well as the consistency of the printing and construct bioassembly procedures. All operators followed the same predefined step‐by‐step workflow developed for 3D slicer, with precise segmentation instructions as explained in the experimental section. Additionally, the operators independently generated the datasets before pooling them. This approach aimed to ensure that the CV values truly reflected inter‐operator variability rather than methodological inconsistencies. The CV ranged from 0.8% to 13% for volume measurements and from 1.3% to 12% for SNR measurements, confirming that these parameters can be reliably extracted using an open‐source platform [24]. These values are consistent with established thresholds, where CV < 10% is considered excellent and 10%–20% acceptable [25]. This finding is particularly important because the lack of standardised, transferable analysis tools remains a major limitation in the field, often preventing meaningful comparisons between studies. By validating a reproducible analysis strategy, our results support the use of MRI not only as an imaging modality but also as a quantitative evaluation tool for bioprinted constructs.

The second objective of this study was to investigate the longitudinal behaviour of contrast‐agent‐labelled cells within multilayer 3D constructs. Previous work has shown that cellular MRI enables tracking of labelled cells both in vitro and in a murine model after LAB, as well as optimising contrast agent concentration and cell density [5]. First, MRI images brought to light interesting insights into the internal architecture of the constructs. Due to the nature of biopaper stacking and the presence of medium inside the bioassembly devices, some constructs appeared not to be completely ‘flat’. Individual layers were not always parallel to each other, and air bubbles as well as structural irregularities could be observed, especially in thicker constructs. The utility of MRI for monitoring these intrinsic architectural differences is particularly relevant for the longitudinal tracking of complex 3D architectures and offers promising perspectives for further research.

Second, as early as 1 day after bioprinting, MRI enabled assessment of printing quality. The initial statistically significant differences in volume on Day 1 between S3 (and S6) versus S9 constructs can be attributed to the number of layers present in each construct.

The difference in volume of labelled cells between three‐ and six‐layer constructs 1 day after the bioprinting could be explained by variations in printing density and droplet formation when using cell‐based bioinks [7, 26]. Because the labelling of the cells by contrast agents was performed in the same manner for all of the printed cells, we can expect that this difference could be due to the heterogeneity inside the bioink layer prior to printing. Because the bioink used in this study was a suspension of living cells, a natural process of sedimentation can occur, even in a short time between bioink preparation and printing. Due to this, a manual resuspension before spreading the bioink on top of a donor slide needs to be performed. Unfortunately, this leaves a possibility of the bioink not being properly resuspended before spreading, leading to some parts of the printed pattern having fewer cells. In addition, other factors, such as small variations in droplet formation during LAB (physical parameters), can contribute to those results. This is consistent with the known variability associated with cell‐based bioinks [7, 26].

On the other hand, the similar SNR values measured at day 1 in S6 and the three S9 constructs may reflect a broadly comparable spatial distribution of labelled cells across these constructs. However, because SPIO‐induced hypointensity may reach a signal floor, similar SNR values cannot be interpreted as evidence of reproducible cell labelling or equivalent intracellular iron loading. The higher SNR values measured in the three‐layer construct at Day 1 can be explained by differences in the MR image acquisition parameters. Indeed, the three‐layer construct was imaged with a lower spatial resolution than S6 and S9, which increases the partial voluming effect in the voxels containing the labelled cells, especially with the surrounding cell medium. This result highlights the importance of high‐spatial resolution protocols to study such 3D constructs, like the ones used in the current study.

Finally, beyond methodological validation, our findings provide insights into the influence of construct architecture on longitudinal MRI‐derived parameters.

The high inter‐operator reliability indicates that the observed temporal changes primarily reflect biological phenomena rather than variability introduced by the analysis workflow. The volume occupied by the labelled cells may reflect either cell spreading occurring during longitudinal monitoring, leading to an increase in the ROI volume, as observed for S6, or cell death, leading to a decrease in the ROI volume, as observed for S9B and S9C. Nevertheless, previous work [27] demonstrated that proliferation of SPIO‐labelled cells may induce progressive dilution of the intracellular label among daughter cells (leading to a decrease in the ROI volume), potentially affecting MRI detectability over time without necessarily implying loss of the cell population.

In parallel, the SNR of labelled cells may be influenced by cell proliferation, leading to dilution of the iron oxide particles and consequently an increase in SNR. However, SNR may also be affected by contrast agent degradation or by cell death, followed by the release and rinsing of the contrast agent during medium renewal. These mechanisms may also contribute to an increase in SNR.

The precise contribution of each mechanism to the evolution of SNR and volume remains difficult to determine in the present study. Additional analyses, particularly regarding in‐depth histological assessment, would be required to further clarify these longitudinal changes.

These differences in the evolution of the ROI volume and SNR between thinner constructs compared to thicker ones could be explained by the effects of nutrient availability, making it clear that thinner constructs are more likely to maintain sufficient nutrient diffusion [28, 29]. On the other hand, more layers increase the effect of cell‐to‐cell signalling, making a thicker construct more beneficial in cell communication [30, 31]. Similar limitations related to diffusion and construct thickness have been reported in 3D hydrogel and bioprinted systems, where increased thickness is associated with heterogeneous cell viability and metabolic activity [32, 33]. However, increased construct thickness may also complicate translation to in vivo applications. That is why three‐layer constructs were implanted into mice in the current study.

The ability of MRI to resolve printed patterns and individual layers over time further highlights its relevance for monitoring spatial fidelity in complex bioprinted architectures. Preservation of spatial organisation is a critical determinant of biological function in engineered tissues, particularly for strategies relying on patterned co‐cultures or guided vascularisation. In this regard, the post‐mortem calvarial defect experiments provide a first proof of concept that MRI can be applied to anatomically relevant settings, offering superior depth penetration and 3D resolution compared to fluorescence imaging. This superiority is supported by the findings of other authors [34, 35].

Other imaging modalities, such as optical coherence tomography (OCT), computed tomography (CT) or photoacoustic imaging, are highly effective for detecting fine structural features in 3D constructs, including layer‐thickness variations and surface irregularities. However, their applicability for in vivo longitudinal tracking remains limited due to restricted penetration depth, signal interference from blood or air and, in the case of CT, exposure to ionising radiation, which precludes repeated imaging [36, 37, 38, 39, 40, 41, 42].

In this study, we used a non‐destructive, non‐irradiative, longitudinal MRI assessment to track SPIO‐labelled constructs over time without damaging the samples, which fully aligns with 3R principles in preclinical research [43]. 3D in vitro models offer viable alternatives for animal experiments [44], whereas longitudinal follow‐up of the same construct generates multiple parameters (voxel count, ROI volume and SNR) [45]. Moreover, MRI enables the tracking of the whole construct without biopsies and histological sampling, minimising animal stress and early detection of complications, consistent with the refinement principle [46].

Despite these promising results, several limitations must be acknowledged. The use of iron oxide–based contrast agents inherently limits the interpretation of MRI signals in terms of cell fate and viability, particularly in vivo, where particle redistribution and macrophage uptake may occur [47, 48]. Previous studies have shown that SPIO‐ or MPIO‐based MRI does not necessarily reflect cell viability, as iron particles may persist after cell death or be taken up by host or surrounding cells [47, 49]. Our results are consistent with these observations and emphasise that MRI‐based cell tracking should be interpreted as a measure of construct localisation and spatial persistence rather than a direct surrogate for long‐term cell survival. In addition to tracking bioprinted SPIO‐labelled cells, MRI also enables evaluation of construct perfusion and vascular integration using dynamic contrast‐enhanced (DCE) protocols. It can quantify parameters such as blood volume, perfusion and microvascular permeability, which have been used in regenerative medicine to assess neovascularisation. Combining cell tracking with DCE‐MRI in future studies could provide additional information on the functional maturation of the surrounding tissues as well as perfusion inside the constructs [50, 51].

MRI approaches based on SPIO or other exogenous contrast agents require additional cell processing and quality control steps before tissue production. This includes incubation of the cells with the labelling agent, removal of unbound particles and verification of labelling efficiency, cell viability, phenotype and functional properties. For routine or large‐scale tissue manufacturing, these additional procedures may increase processing time, introduce batch‐to‐batch variability and complicate the definition of standardised release criteria, thereby limiting scalability and clinical translation. Although preclinical studies have shown that intracellular iron oxide labelling can provide strong MRI contrast with limited short‐term effects on cell viability and differentiation, signal persistence after cell death and label dilution during cell division prevent reliable long‐term quantification of viable cells [49]. Label‐free MRI approaches based on intrinsic tissue contrast could simplify the manufacturing workflow by avoiding exogenous labelling; however, they generally provide lower sensitivity and specificity for tracking individual cell populations [52]. MRI reporter genes may offer an alternative for longitudinal cell monitoring but require genetic modification and raise additional sensitivity, safety and regulatory concerns [53]. These respective advantages and limitations should therefore be considered when translating MRI monitoring to routine tissue production or clinical applications.

The analysis pipeline developed here was designed to be simple, fast and reproducible. We acknowledge that delineation of the labelled cells could be improved by increasing the spatial resolution of the MR images. Ideally, the spatial resolution should be comparable to the size of the blooming effect induced by the SPIO in order to limit partial volume effects. Nevertheless, increasing the spatial resolution would considerably prolong acquisition times, making them incompatible with longitudinal follow‐up of printed cells. In addition, the ROI containing the labelled cells could be manually adjusted to more closely fit the hyposignal generated by the labelled cells. However, this would require the introduction of an additional manual step in the analysis pipeline, potentially leading to increased variability.

Although the 3D slicer protocol developed here demonstrated high reproducibility, with no statistically significant differences between operators, there remains an opportunity to further improve it. Future integration of artificial intelligence–based automated analysis could further improve the reproducibility and reliability of the image analysis methods developed here. Studies have shown that this approach could help in the standardisation of the quantitative analysis and may reduce operator bias [54, 55].

## Conclusion

In this study, we established a reproducible MRI‐based workflow for the non‐destructive longitudinal evaluation of laser‐assisted bioprinted 3D constructs. MRI enabled reliable visualisation of printed cell patterns and multilayer architectures over time, whereas quantitative analysis demonstrated high inter‐operator reproducibility and detected construct‐dependent changes in volume and signal characteristics.

Although iron oxide–based MRI does not directly assess cell viability, it provides robust information on construct localisation, spatial organisation and temporal stability. The successful visualisation of bioprinted constructs in a post‐mortem calvarial defect model further supports the translational potential of this approach.

Together, these findings position MRI as a valuable tool for standardised longitudinal assessment and comparative evaluation of complex bioprinted constructs in tissue engineering.

## Author Contributions

**Mina Medojević:** investigation, writing – original draft, writing – review and editing and visualisation. **Charles Handschin:** investigation, resources, writing – review and editing. **Ayako Washio:** conceptualisation, methodology, resources, writing – review and editing. **Malou Lea:** investigation, resources, writing – review and editing. **Laurence Dallet:** conceptualisation, methodology, writing – review and editing. **Aleksandar Jakovljević:** conceptualisation, methodology, validation, writing – review and editing, supervision. **Emeline J. Ribot:** conceptualisation, methodology, validation, formal analysis, writing – review and editing. **Olivia Kérourédan:** conceptualisation, methodology, validation, formal analysis, writing – review and editing, supervision.

## Funding

This research has been funded by Département STS (R23061), Fondation des Gueules Cassées (35‐2023), Fondation de l'Avenir (AP‐RM‐24B‐036) and Ministry of Science, Technological Development and Innovation of the Republic of Serbia (451‐03‐34/2026‐03/200129 and 451‐03‐33/2026‐03/200129).

## Conflicts of Interest

The authors declare no conflicts of interest.

## Supporting information


**Figure S1:** Example of a workflow to apply Slicer aimed at extracting the volume and signal‐to‐noise ratio of labelled cells from MR images.

## Data Availability

All data are presented in the paper. The data that support the findings of this study are available from the corresponding author upon reasonable request.
